# Outcome of stage IV cancer patients receiving in-hospital cardiopulmonary resuscitation: a population-based cohort study

**DOI:** 10.1038/s41598-019-45977-4

**Published:** 2019-07-01

**Authors:** Meng-Rui Lee, Kai-Lun Yu, Hung-Yang Kuo, Tsung-Hao Liu, Jen-Chung Ko, Jaw-Shiun Tsai, Jann-Yuan Wang

**Affiliations:** 1 Department of Internal Medicine, National Taiwan University Hospital, Hsin-Chu Branch, Hsin-Chu, Taiwan; 2 Department of Internal Medicine, National Taiwan University Hospital, Taipei, Taiwan; 3 Department of Oncology, National Taiwan University Hospital, Taipei, Taiwan; 4 Department of Family Medicine, National Taiwan University Hospital, Taipei, Taiwan; 5 Institute of Epidemiology and Preventive Medicine, College of Public Health, National Taiwan University, Taipei, Taiwan

**Keywords:** Cancer, Outcomes research

## Abstract

The effects of cardiopulmonary resuscitation (CPR) on patients with advanced cancer remain to be elucidated. We identified a cohort of patients with stage-IV cancer who received in-hospital CPR from the Taiwan Cancer Registry and National Health Insurance claims database, along with a matched cohort without cancer who also received in-hospital CPR. The main outcomes were post-discharge survival and in-hospital mortality. In total, 3,446 stage-IV cancer patients who underwent in-hospital CPR after cancer diagnosis were identified during January 2009–June 2014. A vast majority of the patients did not survive to discharge (n = 2,854, 82.8%). The median post-discharge survival was 22 days; 10.1% (n = 60; 1.7% of all patients) of the hospital survivors received anticancer therapy after discharge. We created 1:1 age–, sex–, Charlson comorbidity index (CCI)–, and year of CPR–matched noncancer and stage-IV cancer cohorts (n = 3,425 in both; in-hospital mortality rate = 82.1% and 82.8%, respectively). Regression analysis showed that the stage-IV cancer cohort had shorter post-discharge survival than did the noncancer cohort. The outcome of patients with advanced cancer was poor. Even among the survivors, post-discharge survival was short, with only few patients receiving further anticancer therapy.

## Introduction

Performing cardiopulmonary resuscitation (CPR) on patients with advanced cancer is always a clinical dilemma for clinicians, patients, and their caregivers^1,2^. CPR, if no benefit, causes suffering for patients and psychological trauma for their loved ones. Studies conducted more than a decade ago have indicated that CPR outcome is generally dismal among patients with cancer^3,4^. Even if spontaneous circulation returns, only a small proportion of these patients survive to discharge^3^. In one meta-analysis, metastatic cancer patients receiving in-hospital CPR had only a 5.6% chance of survival to discharge^4^. Nevertheless, a recent multicentre study in France reported a 14% 6-month survival rate among cancer patients with cardiac arrest who were admitted to an intensive care unit (ICU)^5^. In another study, only 5.8% of cancer patients who received CPR during their ICU stay left the hospital alive^6^. The conflicting results obtained by these studies represent a crucial topic worthy of discussion. Although most physicians would agree that cancer patients receiving CPR have a poor prognosis, whether the survival rate of cancer patients receiving CPR changes over time, namely due to improvements in critical and cancer care, remains unclear. Updated epidemiological studies, especially population-based studies, are best placed to answer this question.

Several clinical questions also remain to be answered. First, the clinical course of hospital survivors has not been investigated thoroughly. Little evidence on whether hospital survivors can tolerate further anticancer therapy has been gathered. Moreover, a comparison between the outcomes for cancer and noncancer patients receiving CPR is required. Patients with cancer receiving CPR are generally considered to have poorer outcomes than those without cancer^7^. The answers to the aforementioned research questions would have an impact on medical resource allocation and provide implications for healthcare policymaking. Furthermore, such findings could guide patients and family caregivers in making CPR and do-not-resuscitate (DNR) decisions.

Therefore, we conducted this study to investigate the outcome and prognostic factors in stage-IV cancer patients who received CPR in Taiwan during 2009–2014. To this end, we created a population-based cohort of stage IV cancer patients receiving in-hospital CPR in Taiwan. Also, to provide a general and comparable clinical picture of stage IV cancer patients receiving CPR, we created a matched non-cancer cohort who also received CPR, which was much more commonly encountered in clinical practice, to contrast with the outcome of cancer patients.

## Materials and Methods

### Ethics statement

The Institutional Review Board of National Taiwan University Hospital Hsin-Chu Branch approved this study (NTUH-HC REC: 105-040-E) and waived the need for informed consent because the data utilised in this retrospective study were deidentified.

### Participants and definition

We conducted this study by linking Taiwan National Health Insurance (NHI) claims data, mortality data from the Department of Statistics, and Taiwan Cancer Registry data. The NHI claims data in Taiwan have been previously described^8–10^. In brief, a compulsory universal NHI programme has been implemented by the Bureau of NHI (currently the NHI Administration [NHIA]) since 1995. This programme covers more than 98% of the total Taiwan population (23 million residents). As a single-payer health insurance system, the NHI database administered by the NHIA provides a population-based research platform for epidemiology studies^8–10^.

Launched in 1979, the Taiwan Cancer Registry is a prospective population-based cancer data collection platform. In the registry, initial-diagnosis TNM staging according to the American Joint Committee on Cancer staging edition is available in a long-form database, which contains data on more than 90% of all cancer patients in Taiwan^11^. Researchers can follow cancer patients from their initial diagnosis and treatment course to end of life through linkage between the Taiwan Cancer Registry, NHI claims data, and mortality data.

We first identified patients with incident stage-IV cancer from the Taiwan Cancer Registry; patients with initial diagnoses between 2009 and June 2014 were considered. Patients were included if they received in-hospital CPR after their cancer diagnosis. The hospitalisation course of first in-hospital CPR episode was considered the index hospitalisation. The exclusion criteria were (1) receipt of CPR before stage-IV cancer diagnosis and (2) age at diagnosis < 20 years. The cohort entry date was defined as the admission day of the index hospitalisation. We also identified all patients in Taiwan NHI claims database who received in-hospital CPR between January 2009 and June 2014. The exclusion of patients with cancer was achieved by excluding cancer records in the Taiwan Cancer Registry or the presence of cancer diagnosis (International Classification of Diseases, Ninth Revision, Clinical Modification, ICD-9-CM code: 140-208) in any one of five hospitalisation or three outpatient visit diagnosis codes.

For comparing CPR outcome between patients with stage IV cancer and without any cancer, we also 1:1 matched the stage IV cancer group with a noncancer patient group.

### Definition and data collection

CPR was identified using the procedure code for payment (47029C). In Taiwan, the NHI payment when CPR is performed is calculated in units of 10 min (https://www.nhi.gov.tw). Cancer type was divided into 14 categories according to the International Classification of Diseases for Oncology, 3rd edition (ICD-O-3) codes for each cancer type (Appendix Table 1). The categorization of cancer types also followed previous Taiwan Cancer registry evaluation report^12^. We used the Charlson comorbidity index (CCI) to assess the underlying medical condition of patients and calculated the CCI using NHI claims data in medical records with dates within the 1 year prior to cohort entry^13^. When calculating the CCI of the patients with stage-IV cancer, the cancer-related score and component were not included (cancer-free CCI). Socioeconomic status was determined by income reported for NHI premium calculation, which was divided into low income (receiving government subsidies due to being below the lowest living index and being exempt from NHI premiums and copayment), ≤ Q1, Q1–Q3, and ≥ Q3, as previously detailed^14^.

Primary disease diagnosis was retrieved through the major in-patient diagnosis record of index hospitalisation. The diagnosis codes for categorising the primary disease diagnoses are summarised in Appendix Table 2.

### Statistical analysis

Proportions or means were used to describe the demographic and clinical characteristics of the patients. The standardised difference was used to compare continuous and categorical variables at baseline before the index hospitalisation. The primary outcome was post-discharge survival, which was defined as the interval between the date of discharge and date of death. The secondary outcome was in-hospital mortality. Participants were censored if they were still alive at end of the study period (December 31, 2014).

The stage-IV cancer group was 1:1 matched with a noncancer group—both groups receiving in-hospital CPR—by exactly matching (not propensity score matching) with age, sex, year of CPR, and CCI. Logistic regression was used to identify factors associated with in-hospital mortality. The proportional hazards regression model was applied to explore the factors associated with post-discharge survival. Variables included cancer categories, primary diagnosis for hospitalization, sex, age, CCI score, socioeconomic status, cardioversion, duration of CPR, interval between diagnosis and CPR, chemotherapy, radiotherapy and tyrosine kinase inhibitor. These variables were selected because they potentially had an impact on patient survival^15,16^.

All data analyses were performed using SAS (version 9.4; SAS Institute Inc., Cary, NC, USA). A P of <0.05 on a two-sided test or a standardised difference of >0.1 was considered statistically significant.

## Results

### Patient identification

**Figure 1:**
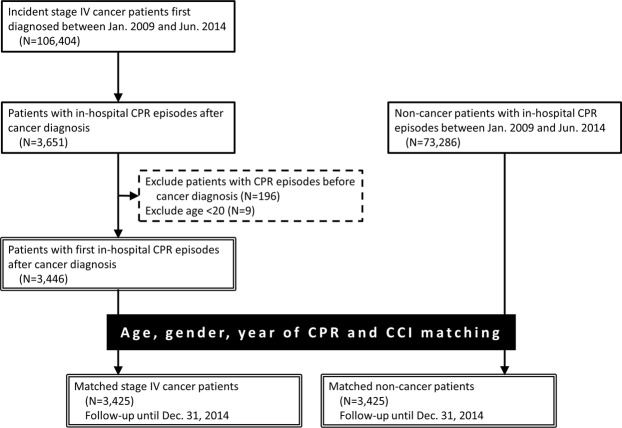
Flowchart of patient recruitment.

The result of the patient identification process is summarised in Fig. 1. For the study period, 3,446 stage-IV cancer patients receiving in-hospital CPR were included.

### Demographic data

The clinical characteristics of the included patients with stage-IV cancer are summarised in Table 1. Among the 3,446 patients, the majority were male (n = 2,545, 73.9%). The most common specific cancer type was lung cancer (n = 1,102, 32.0%), followed by oral cancer (n = 325, 9.4%) and colon cancer (n = 238, 6.9%). The great majority of patients underwent anticancer therapy before they received CPR, with the therapies including chemotherapy (n = 2,173, 63.1%) and radiotherapy (n = 1,595, 46.3%). The mean interval between cancer diagnosis and CPR was 317.1 days (standard deviation [SD] = 388.3 days) and did not differ between the hospital survivors and nonsurvivors (standardised difference [STD] = 0.031).

**Table 1:** Clinical characteristics of Stage IV cancer patients who received in-hospital CPR.

	Overall patients (n = 3,446)	Survived to discharge (n = 592)	Died during hospitalization (n = 2854)	STD
**Age** (mean ± SD)	64.1 ± 14.4	65.0 ± 14.2	64.0 ± 14.5	0.074
**Male**	2545 (73.9)	442 (74.7)	2103 (73.7)	0.016
**Socioeconomic status**				
Low income	150 (4.4)	19 (3.2)	131 (4.6)	0.071
≤Q1	1243 (36.1)	208 (35.1)	1035 (36.3)	0.024
Q1–Q3	1332 (38.7)	244 (41.2)	1088 (38.1)	0.063
>Q3	721 (20.9)	121 (20.4)	600 (21.0)	0.014
**Cancer type**				
Oral Cavity	325 (9.4)	75 (12.7)	250 (8.8)	0.127
Oropharynx	177 (5.1)	39 (6.6)	138 (4.8)	0.076
Hypopharynx	174 (5.1)	38 (6.4)	136 (4.8)	0.072
Esophagus	163 (4.7)	26 (4.4)	137 (4.8)	0.020
Stomach	194 (5.6)	16 (2.7)	178 (6.2)	0.172
Colon	238 (6.9)	43 (7.3)	195 (6.8)	0.017
Rectum	135 (3.9)	27 (4.6)	108 (3.8)	0.039
Liver	237 (6.9)	30 (5.1)	207 (7.3)	0.091
Lung	1102 (32.0)	171 (28.9)	931 (32.6)	0.081
Breast	83 (2.4)	16 (2.7)	67 (2.4)	0.023
Cervix	38 (1.1)	8 (1.4)	30 (1.1)	0.028
Prostate	170 (4.9)	35 (5.9)	135 (4.7)	0.053
Bladder	49 (1.4)	7 (1.2)	42 (1.5)	0.025
Other	361 (10.5)	61 (16.9)	300 (10.5)	0.007
**CPR year**				
2009	579 (16.8)	108 (18.2)	471 (16.5)	0.046
2010	656 (19.0)	117 (19.7)	539 (18.9)	0.022
2011	655 (19.0)	116 (19.6)	539 (18.9)	0.018
2012	609 (17.7)	108 (18.2)	501 (17.6)	0.018
2013	568 (16.5)	88 (14.9)	480 (16.8)	0.054
2014	379 (11.0)	55 (9.3)	324 (11.4)	0.068
**CCI** (mean ± SD)	3.90 ± 2.20	4.12 ± 2.31	3.85 ± 2.17	0.119
**Anti-cancer therapy**	2553 (74.1)	430 (72.6)	2123 (74.4)	0.040
Chemotherapy	2175 (63.1)	361 (70.0)	1814 (63.6)	0.053
Radiotherapy	1595 (46.3)	285 (48.1)	1310 (45.9)	0.045
TKI	316 (9.2)	52 (8.8)	264 (9.3)	0.016
**Interval between cancer diagnosis and index admission** (mean ± SD)	317.1 ± 388.3	327.1 ± 395.8	315.0 ± 386.8	0.031
**Primary disease for admission**				
Cancer-related	2325 (67.5)	380 (64.2)	1945 (68.2)	0.082
Cardiovascular	117 (3.4)	22 (3.7)	95 (3.3)	0.021
Gastrointestinal	77 (2.2)	8 (1.4)	69 (2.4)	0.078
Neurologic	20 (0.6)	7 (1.2)	13 (0.5)	0.081
Renal	19 (0.6)	4 (0.7)	15 (0.5)	0.019
Respiratory	476 (13.8)	100 (16.9)	376 (13.2)	0.104
Sepsis	23 (0.7)	3 (0.5)	20 (0.7)	0.025
Trauma	17 (0.5)	5 (0.8)	12 (0.4)	0.054
Other	372 (10.8)	63 (10.6)	309 (10.8)	0.006
**Receiving cardioversion**	613 (17.8)	114 (19.3)	499 (17.5)	0.046
**CPR duration (minutes)**	23 ± 17	17 ± 13	24 ± 18	0.409

Note: CCI, charlson comorbidity index; CPR, cardiopulmonary resuscitation; SD, standard deviation; STD, standardized difference; TKI, tyrosine kinase inhibitor.

Data are number (%) unless otherwise mentioned.

Percentages in the three columns are for the column and not the row.

The primary disease diagnosis of index hospitalisation was cancer related in the majority of patients (n = 2,325, 67.5%), followed by respiratory disease related (n = 476, 13.8%). During the CPR episode, 17.8% of the patients received cardioversion. CPR generally lasted 20–30 min (mean of 2.3 ± 1.7 in units of 10 min). Only 17.2% of patients (n = 592) survived the index hospitalisation.

Those who survived to discharge were more likely to have oral cancer, admission due to respiratory disease, shorter CPR duration, and higher CCI, but less likely to have stomach cancer.

The overall in-hospital mortality rate was 82.8% (n = 2,854), which increased to 87.5% (n = 2,996) if hospital survivors were defined as being alive 7 days after discharge. The in-hospital mortality rate by year and with two different definitions is illustrated in Appendix Fig. 1.

### Factors associated with in-hospital mortality

Multivariable logistic regression revealed that stomach cancer (adjusted odds ratio [aOR] = 2.61, 95% confidence interval [CI] = 1.44–4.75), liver cancer (aOR = 1.79, 95% CI = 1.09–2.95), and longer CPR duration (aOR = 1.33, 95% CI = 1.24–1.43 per 10-min increment) were associated with higher in-hospital mortality rate, whereas oral cancer (aOR = 0.66, 95% CI = 0.44–0.99) was associated with lower in-hospital mortality rate. Furthermore, the proportion of in-hospital mortality and survival of individual cancer type with two definitions was described in Appendix Table 3.

### Hospital survivors among stage-IV cancer patients receiving in-hospital CPR

**Figure 2:**
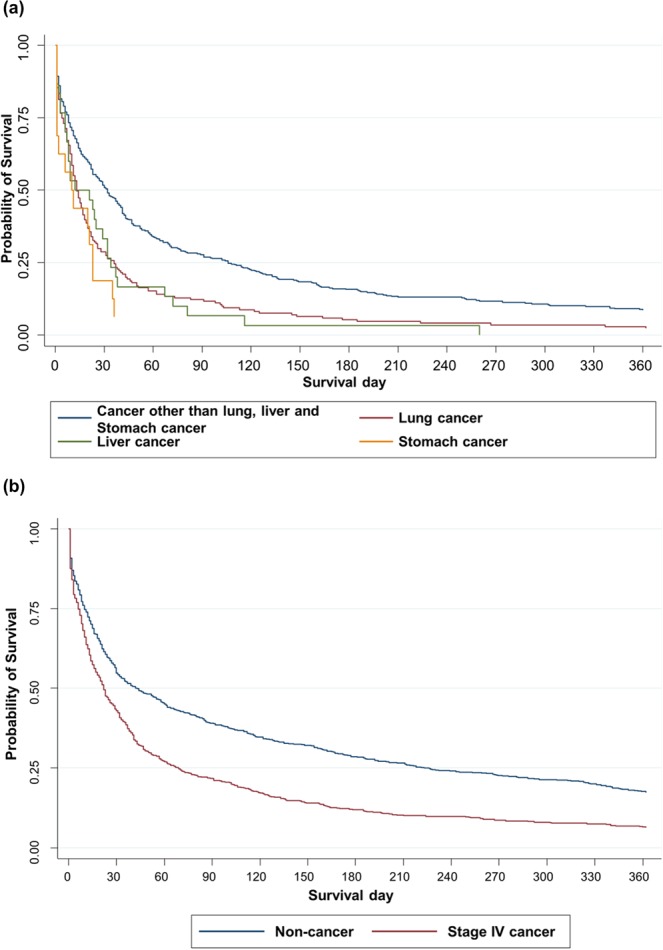
Post-discharge survival curves of liver cancer, stomach cancer, lung cancer and other cancer (**a**), and matched non-cancer and stage IV cancer patients (**b**).

Among the 592 hospital survivors, only a small proportion received further anticancer therapy (chemotherapy, n = 50, 8.5%; radiotherapy, n = 32, 5.4%; total, n = 60, 10.1%). The median post-discharge survival was 22 days. The survival curves of different cancer types are presented in Fig. 2a.

### Factors associated with post-discharge survival

The risk factors associated with shorter post-discharge survival were stomach cancer (adjusted hazard ratio [aHR] = 3.21, 95% CI = 1.72–5.98), liver cancer (aHR = 2.34, 95% CI = 1.46–3.76), lung cancer (aHR = 1.78, 95% CI = 1.30–2.46), receipt of chemotherapy prior to CPR (aHR = 1.33, 95% CI = 1.09–1.63), CPR in 2014 (compared with that in 2009, aHR = 2.03, 95% CI = 1.41–2.91), and longer CPR duration (aHR = 1.15, 95% CI = 1.08–1.23 per 10-min increment). Patients who were admitted due to renal disease (aHR = 0.32, 95% CI = 0.11–0.92) and trauma (aHR = 0.30, 95% CI = 0.11–0.81) had better prognosis. The prognostic factors associated with in-hospital mortality and post-discharge survival are summarised in Table 2.

**Table 2:** Independent prognostic factors of in-hospital mortality and post-discharge survival.

	In-hospital mortality (logistic regression)			Post-discharge survival (proportional hazards regression)		
	Adjusted OR*	95% CI	P value	Adjusted HR*	95% CI	P value
CPR year in 2014 (compared with 2009)	1.47	1.01–2.16	0.129	2.03	1.41–2.91	<0.001
Stomach cancer	2.61	1.44–4.75	0.002	3.21	1.72–5.98	<0.001
Liver cancer	1.79	1.09–2.95	0.022	2.34	1.46–3.76	<0.001
Lung cancer	1.35	0.95–1.92	0.096	1.78	1.30–2.46	<0.001
Oral cancer	0.66	0.44–0.99	0.043	0.81	0.56–1.15	0.239
CCI	0.93	0.87–0.99	0.023	0.94	0.88–1.00	0.062
CPR duration (every ten minutes)	1.33	1.24–1.43	<0.001	1.15	1.08–1.23	<0.001
Chemotherapy prior to CPR	1.10	0.88–1.36	0.404	1.33	1.09–1.63	0.004
Admission for renal disease	0.66	0.21–2.12	0.485	0.32	0.11–0.92	0.034
Admission for trauma disease	0.50	0.17–1.50	0.214	0.30	0.11–0.81	0.018

Note: CCI, charlson comorbidity index; CI, confidence interval; CPR, cardiopulmonary resuscitation**;** HR, hazard ratio; OR, odds ratio.

*Variables in the model included year of CPR, cancer types, primary disease for admission, gender, age, socioeconomic status, cardioversion, radiotherapy prior to CPR, chemotherapy prior to CPR, and cancer diagnosis to CPR.

### Matched cohorts of stage-IV cancer and noncancer patients receiving in-hospital CPR

A stage-IV cancer group and 1:1 age–, sex–, year of CPR–, and CCI–matched comparison group of noncancer patients (Table 3) were selected (each with 3,425 patients; 21 stage-IV cancer patients could not be matched with noncancer patients). 589 cancer and 612 non-cancer patients survived to discharge (Appendix Table 4). The mortality rate was 82.8% (n = 2,836) and 82.1% (n = 2,813) in stage-IV cancer and noncancer cohorts, respectively (STD = 0.039, p = 0.46). Logistic regression revealed that no association between noncancer and stage-IV cancer cohorts and in-hospital mortality. Proportional hazard ratio analysis revealed that the patients with stage-IV cancer had shorter post-discharge survival (aHR = 1.28, 95% CI = 1.08–1.50) than the noncancer patients. The post-discharge survival curves of the matched noncancer and stage-IV cancer groups are presented in Fig. 2b.

**Table 3:** Clinical characteristics of matched stage IV cancer and non-cancer patients who received in-hospital CPR (21 stage-IV cancer patients could not be matched with noncancer patients and were therefore excluded from this analysis).

	Cancer patients (n = 3425)	Non-cancer patients (n = 3425)	STD
**Age** (mean ± SD)	64.3 ± 14.3	64.3 ± 14.3	0
**Male**	2536 (74.0)	2536 (74.0)	0
**Socioeconomic status**			
Low income	147 (4.3)	208 (6.1)	0.080
≤Q1	1236 (36.1)	1371 (40.0)	0.081
Q1–Q3	1323 (38.6)	1244 (36.3)	0.048
>Q3	719 (20.1)	602 (17.6)	0.087
**CPR year**			
2009	577 (16.9)	577 (16.9)	0
2010	655 (19.1)	655 (19.1)	0
2011	651 (19.0)	651 (19.0)	0
2012	603 (17.6)	603 (17.6)	0
2013	563 (16.4)	563 (16.4)	0
2014	376 (11.0)	376 (11.0)	0
**CCI** (mean ± SD)	3.89 ± 2.19	3.89 ± 2.19	0
**Primary Disease for Admission**			
Cancer-related	2312 (67.5)	0	2.038
Cardiovascular	116 (3.4)	742 (21.7)	0.575
Gastrointestinal	77 (2.3)	209 (6.1)	0.194
Neurologic	20 (0.6)	243 (7.1)	0.344
Renal	19 (0.6)	50 (1.5)	0.091
Respiratory	473 (13.8)	920 (26.9)	0.329
Sepsis	22 (0.6)	76 (2.2)	0.133
Trauma	17 (0.5)	240 (7.0)	0.348
Other	369 (10.8)	945 (27.6)	0.437
**Receiving cardioversion**	607 (17.7)	830 (24.2)	0.160
**CPR duration (10 minutes)**	2.3 ± 1.7	2.5 ± 2.0	0.132

Note: CCI, charlson comorbidity index; CPR, cardiopulmonary resuscitation; SD, standard deviation; STD, standardized difference; TKI, tyrosine kinase inhibitor.

Data are number (%) unless otherwise mention.

## Discussion

We have noted that patients with stage-IV cancer who received CPR had poor prognosis, with lung, liver, or stomach cancer patients having even poorer outcomes. The median survival after discharge was less than 1 month, and few survivors received subsequent anticancer treatment after their CPR event. Although the in-hospital mortality rates of the stage-IV cancer and noncancer cohorts were similar, the post-discharge survival among the patients with stage-IV cancer and receiving in-hospital CPR was inferior to that of the noncancer patients receiving CPR. Thus, the decision of whether to perform CPR on those with advanced cancer must be carefully justified.

In Taiwan, end-stage cancer patients will receive CPR if no DNR orders has been signed^17^. Discussing CPR issues—widely considered mandatory in the care of patients with late-stage cancer—can be difficult and challenging. Taiwan has an Eastern culture, in which talking about death is taboo^18^. In addition, family caregivers are more frequently involved in decision-making than in Western countries^17^. Inadequate discussion and disagreement between patients and caregivers, however, are common in Taiwan^19^. In one study conducted in Taiwan, DNR orders were almost twice more likely to be signed by surrogates than by patients; a DNR order signed by the patient was associated with higher quality of end of life care^20^. Breaking these communication barriers to achieve better patient care is therefore vital. Strategies such as promoting cultural change to make care more patient-centred, establishing standards for DNR discussions, and improving physician communication skills have been proposed for achieving superior patient care^21^. Our study offers a key message to family caregivers, patients, and physicians in-charge that under most circumstances, refusing to sign a DNR and declining palliative care in late-stage cancer when experiencing cardiac arrest can lead to patient suffering.

Studies on CPR in patients with advanced cancer have reported a 5.6%–15% CPR success rate^4,22,23^. Our study reported a 17.2% in-hospital survival rate and 12.5% survival rate at 7 days post-discharge. While there was no data regarding proportion of patients who underwent withdrawal of life-sustaining treatment post CPR, survival rate may be affected by characteristics of medical care system. The Taiwan NHI is known for its low-cost and comprehensive coverage^24,25^. For instance, patients undergoing prolonged mechanical ventilation (intubation for more than 2 months) can reside in long-term respiratory care facilities under NHI coverage, regardless of underlying disease status and projected survival^26^. For patient families and caregivers, access to medical and nursing care with low financial burden leads to an aggressive attitude towards maintaining patients’ lives^27^.

A Taiwanese study, conducted using a random sample from 5% of the overall population sample in the NHI database, also investigated the outcome of patients receiving CPR between 1997 and 2004^28^. The study reported an overall 11.6% CPR success rate, as defined by surviving to discharge^28^. Comparing with this previous study, our methodology was different^28^. We targeted stage-IV cancer (staging would be unknown without linkage to the cancer registry), linked mortality statistics for definite death date, used whole population dataset and investigated the post-discharge outcome among hospital survivors. Furthermore, we used procedure codes rather than diagnosis codes to identify CPR episodes^28^.

CPR in patients with advanced cancer may extend beyond hospital survival. Survival after discharge is a crucial outcome measure. Furthermore, oncologists should be concerned about whether those who survive until discharge can tolerate or receive further anticancer treatment. Most studies have failed to address these two critical questions^6,29,30^. Here, we found that only approximately 10% of hospital survivors received further anticancer therapy. Intolerance to subsequent anticancer therapy may reflect the devastating nature of cardiac arrest events. Deterioration of neurological function and organ damage possibly precludes the receipt of anticancer therapy^31,32^. If the decision to perform CPR is based on the expectation that further anticancer therapy will be administered, our results suggest that this goal is unachievable in the vast majority of patients.

Our study also revealed that cancer type was an essential prognostic factor for cancer patients who experience cardiac arrest. This may be unsurprising given that the outcome and survival of different cancer types vary, regardless of CPR events^33^. For instance, lung cancer is the leading cause of cancer-related death worldwide, and patients with advanced lung cancer generally have poor prognosis^34^. The treatment options for nonresectable advanced hepatocellular carcinoma are limited^35^. In Taiwan, oral cancer had the highest stage-specific survival, whereas liver cancer had the lowest^36^. Therefore, the presence of advanced cancer and specific cancer type should be considered when discussing CPR decisions with patients and their families.

Some other findings of our study are also of interest. Pre-CPR chemotherapy was associated with shorter post-discharge survival in our study. This may indicate that patients who have previously undergone chemotherapy have less available treatment options if they survived their CPR episode or were in poorer general condition compared with those untreated. In addition, CCI appears to be a favourable prognostic factor for in-hospital mortality. The patients with higher CCI were more likely to receive CPR due to an underlying comorbidity, which can be readily treated, rather than cancer-related complications. This survival benefit, however, was not detected in the post-discharge survival analysis.

Our study included a matched comparison group of noncancer patients, which was uncommon in previous studies^23,33^. The creation of matched comparison cohort of non-cancer patients, however, was not intended to provide a direct comparison of the outcome of cancer and non-cancer patients receiving CPR. These two groups were still different in their primary diagnosis for admission and CPR-related characteristics (Table 3). Rather, we aimed to provide more information to help facilitate decision-making for stage IV cancer patients while CPR for non-cancer patients was more frequently encountered and experienced in clinical practice. Interestingly, despite an approximate one month difference in median survival between cancer and non-cancer patients, there was a long tail of survival curve among both groups. This highlights the fact that for non-cancer patients who experienced CPR events, some may achieve remarkable long-term survival. This was also the case for stage IV cancer patients. Certain cancer types with favorable prognostic factors, such as lung adenocarcinoma with tyrosine kinase inhibitor (TKI)-sensitive epidermal growth factor receptor (EGFR) mutation or hormone receptor-positive breast cancer, may achieve long-term survival once they survived the CPR events^37,38^.

One, however, needs to avoid simply interpreting the results of our study to be that CPR in cancer patients is futile. While survival rate of less than 1% is commonly regarded as the threshold of medical futility^39,40^ and the in-hospital mortality rate was 17.2% in our study. Furthermore, a recent review also addressed that physicians should avoid withdrawal of care in the absence of definite prognostic signs either during or after cardiac arrest^41^. Our study intended to describe the general outcome of advanced cancer patients receiving CPR and thus provided information that could help decision-making. Individual patient evaluation and discussion are still irreplaceable.

Our study has some limitations. First, we could not collect performance status and neurological statuses, both of which are crucial components for understanding prognosis and thus prerequisites for decision-making regarding CPR^42,43^. This may be a direction for future investigations. Second, we could not identify the cause of CPR from the claims database. Nonetheless, we detected no difference between groups with different primary diagnoses at admission.

## Conclusion

Our nationwide population-based study revealed that advanced cancer patients receiving CPR had a poor prognosis, with those having lung, liver, or stomach cancer having even poorer outcome. Even among the hospital survivors, only a small minority went on to receive further anticancer therapy. Given the high in-hospital mortality rate and short survival time among the hospital survivors, strong indications of a high likelihood of survival (e.g. using the most highly effective and tolerable anticancer treatment available) are required to justify the decision to perform CPR on patients with advanced cancer.

## Supplementary information

Appendix Tables and Figure

## Data Availability

All data were deposited in the national health insurance databases located in the Ministry of Health and Welfare, Taiwan and were not available for sharing without permission.
