# Supplementary material for: Outcome of stage IV cancer patients receiving in-hospital cardiopulmonary resuscitation: a population-based cohort study
Source: Sci Rep. 2019 Jul 1;9:9478. doi: 10.1038/s41598-019-45977-4 (PMC6602946; doi:10.1038/s41598-019-45977-4)

**Outcome of stage IV cancer patients receiving in-hospital cardiopulmonary resuscitation: a population-based cohort study**

Meng-Rui Lee, MD^1,2,3^; Kai-Lun Yu, MD^1,2^; Hung-Yang Kuo, MD^1, 4^; Tsung-Hao Liu, MD^1, 4^; Jen-Chung Ko, MD, PhD^1,2^; Jaw-Shiun Tsai, MD, PhD^5^; Jann-Yuan Wang, MD, PhD^2^;

^1^Department of Internal Medicine, National Taiwan University Hospital, Hsin-Chu Branch, Hsin-Chu, Taiwan

^2^Department of Internal Medicine, Department of Oncology^4^ and Department of Family Medicine^5^, National Taiwan University Hospital, Taipei, Taiwan

^3^Institute of Epidemiology and Preventive Medicine, College of Public Health, National Taiwan University

**Appendix Table 1. International Classification of Diseases for Oncology, 3^rd^ edition (ICD-O-3) codes for definition of cancer type**

**Appendix Table 2. Diagnosis code for primary disease diagnosis**

**Appendix Table 3. Percentage of hospital mortality under two definitions among different cancer types**

**Appendix Table 4. Clinical characteristics of matched stage IV cancer and non-cancer patients who received in-hospital CPR and survived to discharge**

**Appendix** **Figure 1. In-hospital mortality rate by year**

**Appendix Table 1. International Classification of Diseases for Oncology, 3^rd^ edition (ICD-O-3) codes for definition of cancer type**

| Cancer Type | ICD-O-3 code |
| --- | --- |
| Oral cavity cancer | C000-C009,C020-C023,C028-C029,C030-C031,C039,C040-C041,C048-C049,C050,C058-C059,C060-C062,C068-C069 |
| Oropharynx cancer | C019,C024,C051,C052,C090-C091,C098-C099,C100-C104,C108-C109,C142,C148 |
| Hypopharynx cancer | C129, C130-C132, C138-C139, C140 |
| Esophageal cancer | C150-C155, C158-C159 |
| Stomach cancer | C160-C166,C168,C169 |
| Colon cancer | C180-C189 |
| Rectum cancer | C199,C209 |
| Liver cancer | C220, C221 |
| Lung cancer | C340-C343,C348-C349 |
| Breast cancer | C500-C509 |
| Cervical cancer | C530-C531, C538-C539 |
| Prostate cancer | C619 |
| Bladder cancer | C670-C679 |
| Other* | C021, C112, C119, C541, C569, C778, C779 |

*Number of patients >3

**Appendix Table 2. Diagnosis code for primary disease diagnosis**

| **Diagnosis** | **International Classification of Diseases, Ninth Revision, Clinical Modification (ICD-9-CM) codes** |
| --- | --- |
| **Cancer-related** | 140-208 |
| **Cardiovascular Disease** |  |
| Myocardial infarction | 410 |
| Coronary atherosclerosis, ischemic heart disease | 411, 414 |
| Paroxysmal ventricular tachycardia, atrial flutter, atrial fibrillation, cardiac arrest, cardiac arrhythmia | 427 |
| Malignant hypertension, Heart failure | 402, 428 |
| Aortic aneurysm, aortic dissection | 441 |
| AV block, bundle branch block | 426 |
| Arterial embolism and thrombosis | 444 |
| Intestine vascular disease | 557 |
| Angina | 413 |
| Mitral valve disease | 394, 396 |
| Cardiomyopathy | 425 |
| Artery and venous anomalies | 747 |
| **Respiratory Disease** |  |
| Respiratory failure | 518 |
| Pneumonia | 482, 486 |
| Pneumonitis | 507 |
| Chronic obstructive pulmonary disease, asthma | 491, 493, 496 |
| Pulmonary hemorrhage | 770.3 |
| Empyema | 510 |
| Pulmonary tuberculosis | 11 |
| Pneumothorax | 512 |
| Pulmonary embolism | 415 |
| Pleuritis | 511 |
| **Sepsis Disease** |  |
| Septicemia | 38 |
| Urinary tract infection, pyelonephritis | 599.0, 590 |
| Peritonitis | 567 |
| Cellulitis and abscess | 682 |
| Acute appendicitis | 540 |
| Endocarditis | 421 |
| Cholangitis | 576.1 |
| Liver abscess | 572.0 |
| **Gastrointestinal Disease** |  |
| Peptic ulcer | 531, 532, 533 |
| Cirrhosis | 571 |
| Gallbladder and common bile duct stone | 574 |
| Hemorrhage of gastrointestinal tract | 578 |
| Hepatic coma, sequelae of chronic liver disease | 572.1, 572.2, 572.3, 572.4, 572.8 |
| Intestinal obstruction, ileus, volvulus, intussusception | 560 |
| Acute pancreatitis | 577 |
| Acute cholecystitis | 575 |
| Esophageal ulcer | 530 |
| Diverticulitis | 562 |
| **Neurologic Disease** |  |
| Intra-cranial hemorrhage | 431, 432 |
| Ischemic stroke | 433, 434 |
| Subarachnoid hemorrhage | 430 |
| Epilepsy | 345 |
| Encephalopathy | 348 |
| Cerebrovascular disease | 436, 437 |
| **Renal Disease** |  |
| Acute renal failure | 584 |
| End-stage renal disease | 585 |
| Intoxication | 969, 967, 989 |
| Acidosis, electrolyte abnormality | 276 |
| Hypertensive renal disease | 403 |
| **Trauma Disease** |  |
| Fracture | 800-829 |
| Cervical dislocation, cervical trauma | 839, 952 |
| Internal organ trauma | 850-854, 860-869 |

**Appendix Table 3. Percentage of hospital mortality under two definitions among different cancer types**

|  | Oral cavity | Oropharynx | Hypopharynx | Esophagus | Stomach | Colon | Rectum | Liver | Lung | Breast | Cervix | Prostate | Bladder | Other |
| --- | --- | --- | --- | --- | --- | --- | --- | --- | --- | --- | --- | --- | --- | --- |
| N (%) of all cancer patients | 325 (9.4) | 177 (5.1) | 174 (5.1) | 163 (4.7) | 194 (5.6) | 238 (6.9) | 135 (3.9) | 237 (6.9) | 1102 (32.0) | 83 (2.4) | 38 (1.1) | 170 (4.9) | 49 (1.4) | 361 (10.5) |
|  |  |  |  |  |  |  |  |  |  |  |  |  |  |  |
| Mortality during hospitalization | 76.9% | 78.0% | 78.2% | 84.1% | 91.8% | 81.9% | 80% | 87.3% | 84.5% | 80.7% | 79.0% | 79.4% | 85.7% | 83.1% |
|  |  |  |  |  |  |  |  |  |  |  |  |  |  |  |
| Mortality during hospitalization and within 7 days after discharge | 80.6% | 83.1% | 81% | 91.4% | 95.4% | 87.0% | 85.9% | 91.6% | 90% | 89.2% | 86.8% | 82.4% | 91.8% | 87.0% |

**Appendix Table 4.** Clinical characteristics of matched stage IV cancer and non-cancer patients who survived to discharge after in-hospital CPR

|  | Cancer  (n=589) | Non-cancer  (n=612) | STD |
| --- | --- | --- | --- |
| **Age** (mean±SD) | 65.1±14.2 | 66.5±13.4 | 0.105 |
| **Male** | 441 (74.9) | 430 (70.3) | 0.103 |
| **Socioeconomic status** |  |  |  |
| Low income | 19 (3.2) | 48 (7.8) | 0.203 |
| ≤Q1 | 208 (35.3) | 271 (44.3) | 0.184 |
| Q1-Q3 | 242 (41.1) | 194 (31.7) | 0.196 |
| >Q3 | 120 (20.4) | 99 (16.2) | 0.109 |
| **CPR year** |  |  |  |
| 2009 | 108 (18.3) | 107 (17.5) | 0.022 |
| 2010 | 117 (19.9) | 128 (20.9) | 0.026 |
| 2011 | 116 (19.7) | 108 (17.7) | 0.053 |
| 2012 | 107 (18.2) | 115 (18.8) | 0.016 |
| 2013 | 86 (14.6) | 99 (16.2) | 0.044 |
| 2014 | 55 (9.3) | 55 (9.0) | 0.012 |
| **CCI** (mean ±SD) | 4.10±2.27 | 4.35±2.20 | 0.109 |
| **Primary Disease for Admission** |  |  |  |
| Cancer-related | 378 (64.2) | 0 | 1.893 |
| Cardiovascular | 22 (3.7) | 121 (19.8) | 0.514 |
| Gastrointestinal | 8 (1.4) | 30 (4.9) | 0.205 |
| Neurologic | 7 (1.2) | 54 (8.8) | 0.356 |
| Renal | 4 (0.7) | 11 (1.8) | 0.101 |
| Respiratory | 99 (16.8) | 225 (36.8) | 0.463 |
| Sepsis | 3 (0.5) | 16 (2.6) | 0.170 |
| Trauma | 5 (0.9) | 22 (3.6) | 0.187 |
| Other | 63 (32.1) | 133 (21.7) | 0.303 |
| **Receiving cardioversion** | 113 (19.2) | 150 (24.5) | 0.129 |
| **CPR duration (10 minutes)** | 1.7±1.3 | 1.9±1.9 | 0.139 |

**NOTE.** CCI, charlson comorbidity index; CPR, cardiopulmonary resuscitation; SD, standard deviation; STD, standardized difference; TKI, tyrosine kinase inhibitor Data are number (%) unless otherwise mention

**Appendix** **Figure 1. In-hospital mortality rate by year**


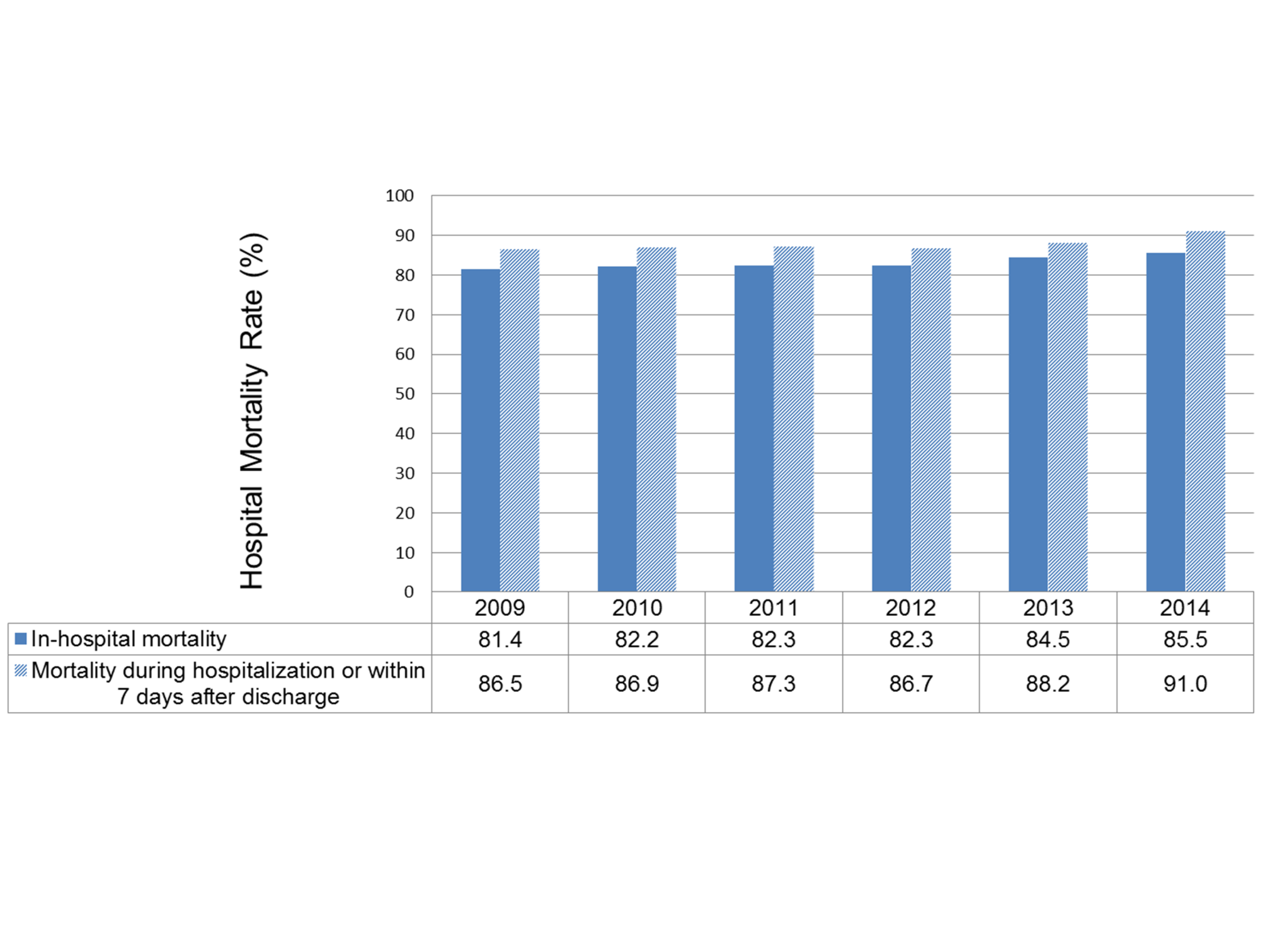

Supplement: Supplementary file 1 — Appendix Tables and Figure [file 41598_2019_45977_MOESM1_ESM.docx]
